# *Toxoplasma gondii* exposure in patients suffering from mental and behavioral disorders due to psychoactive substance use

**DOI:** 10.1186/s12879-015-0912-1

**Published:** 2015-04-03

**Authors:** Cosme Alvarado-Esquivel, David Carrillo-Oropeza, Sandy Janet Pacheco-Vega, Jesús Hernández-Tinoco, Misael Salcedo-Jaquez, Luis Francisco Sánchez-Anguiano, María Nalleli Ortiz-Jurado, Yesenia Alarcón-Alvarado, Oliver Liesenfeld, Isabel Beristain-García

**Affiliations:** Biomedical Research Laboratory, Faculty of Medicine and Nutrition, Juárez University of Durango State, Avenida Universidad S/N, 34000 Durango, Mexico; Hospital of Mental Health “Dr. Miguel Vallebueno”, Secretary of Health, Avenida Fidel Velázquez S/N, 34234 Durango, Mexico; Institute for Scientific Research “Dr. Roberto Rivera-Damm”, Juárez University of Durango State, Avenida Universidad S/N, 34000 Durango, Mexico; Faculty of Nursing and Obstetrics, Juárez University of Durango State, Avenida Cuauhtémoc 223, 34000 Durango, Mexico; Institute for Microbiology and Hygiene, Campus Benjamin Franklin, Charité Medical School, Hindenburgdamm 27, D-12203 Berlin, Germany; Current address: Chief Medical Officer, Medical and Scientific Affairs, Roche Molecular Systems, Pleasanton, CA 94588 USA

**Keywords:** *Toxoplasma gondii*, Infection, Seroprevalence, Psychiatric patients, Psychoactive drug Abuse, Epidemiology, Mexico

## Abstract

**Background:**

*Toxoplasma gondii* infection has been associated with psychiatric diseases. However, there is no information about the link between this infection and patients with mental and behavioral disorders due to psychoactive substance use.

**Methods:**

We performed a case-control study with 149 psychiatric patients suffering from mental and behavioral disorders due to psychoactive substance use and 149 age- and gender-matched control subjects of the general population. We searched for anti-*T. gondii* IgG and IgM antibodies in the sera of participants by means of commercially available enzyme-linked immunoassays. Seroprevalence association with socio-demographic, clinical and behavioral characteristics in psychiatric patients was also investigated.

**Results:**

Anti-*T. gondii* IgG antibodies were present in 15 (10.1%) of 149 cases and in 14 (9.4%) of 149 controls (*P* = 1.0). Anti-*T. gondii* IgM antibodies were found in 11 (7.4%) of the 149 cases and in 16 (10.7%) of the 149 controls (*P* = 0.31). No association of *T. gondii* exposure with socio-demographic characteristics of patients was found. Multivariate analysis of clinical and behavioral characteristics of cases showed that *T. gondii* seropositivity was positively associated with consumption of opossum meat (OR = 10.78; 95% CI: 2.16-53.81; *P* = 0.003) and soil flooring at home (OR = 11.15; 95% CI: 1.58-78.92; *P* = 0.01), and negatively associated with suicidal ideation (OR = 0.17; 95% CI: 0.05-0.64; *P* = 0.008).

**Conclusions:**

Mental and behavioral disorders due to psychoactive substance use do not appear to represent an increased risk for *T. gondii* exposure. This is the first report of a positive association of *T. gondii* exposure with consumption of opossum meat. Further studies to elucidate the role of *T. gondii* infection in suicidal ideation and behavior are needed to develop optimal strategies for the prevention of infection with *T. gondii*.

## Background

*Toxoplasma gondii* infection is a common zoonotic infection all around the world [1]. Infection with *T. gondii* is usually acquired by ingesting water or food contaminated with parasite oocysts shed by cats or by ingestion of raw or undercooked meat containing parasite tissue cysts [2]. Less commonly, *T. gondii* infection has been associated with blood transfusion [3] or transplantation [4,5]. Although most *T. gondii* infected people shows no symptoms, some infected individuals may develop toxoplasmosis, which is a disease with involvement of eyes, lymph nodes and central nervous system [1,2,6]. Toxoplasmosis in immunocompromised patients may be a life-threatening disease [2,7]. In addition, congenital toxoplasmosis may occur after primary *T. gondii* infection during pregnancy [2,8,9].

Infection with *T. gondii* has been associated with some psychiatric disorders including schizophrenia [10-12], personality disorders [13], and obsessive-compulsive disorder [14]. However, the association of *T. gondii* infection with mental and behavioral disorders due to psychoactive substance use has not been studied. In a previous descriptive study in psychiatric patients, we found that 4 of 26 patients with mental and behavioral disorders due to psychoactive substance use had *T. gondii* antibodies [10]. However, the study design (cross-sectional) and the small sample size did not allow us properly assessing the association of infection and disease. Therefore, in the present study, we sought to determine the association of *T. gondii* infection with patients suffering from mental and behavioral disorders due to psychoactive substance use attended in the psychiatric hospital of Durango City, Mexico. Furthermore, the association of *T. gondii* infection with the socio-demographic, clinical and behavioral characteristics of the patients was also investigated.

## Methods

### Study design and study populations

We performed a case-control seroprevalence study in 149 patients suffering from mental and behavioral disorders due to psychoactive substance use (cases) and 149 people from the general population (controls) in Durango City, Mexico from November 2013 to August 2014. Inclusion criteria for the cases were patients suffering from mental and behavioral disorders due to psychoactive substance use attended in the Hospital of Mental Health “Dr. Miguel Vallebueno” in Durango City, aged 18 years and older, and who voluntarily accepted to participate in the study. Gender was not a restrictive criterion for enrollment. Cases were 18-67 (mean = 36.01 ± 12.48) years old, and included 123 males and 26 females. Mental and behavioral disorders in the 149 patients studied were due to use of alcohol (F10) in 38 patients, use of cannabinoids (F12) in 8, use of sedative hypnotics (F13) in 7, use of cocaine (F14) in 1, use of other stimulants including caffeine (F15) in 3, use of tobacco (F17) in 10, and multiple drugs use and use of other psychoactive substances (F19) in 82. Exclusion criterion for the cases was presence of severe illness that impairs their decision to participate in the study. Control subjects were randomly selected from the general population in Durango City. Controls were recruited from homes, schools, work places and streets as previously described [15,16]. Controls were matched with cases by age (±1 year) and gender. Controls were 18-67 (mean = 36.03 ± 12.49) years old, and included 123 males and 26 females. There was no difference in age between cases and controls (*P* = 0.98).

### Socio-demographic, clinical, and behavioral data in cases

Patients submitted a standardized questionnaire in order to obtain their socio-demographic, clinical, and behavioral characteristics. Socio-demographic data obtained included age, gender, birthplace, residence, educational level, occupation, and socioeconomic status. Items of clinical characteristics were health status, history of lymphadenopathy, blood transfusions, transplantation and surgeries, presence of frequent headache, dizziness, and impairments in vision, hearing, memory and reflexes. In female patients, obstetric history was also obtained. In addition, the area of attention (outpatients or inpatients), the psychiatric diagnosis, evolution time, presence of treatment, response to treatment, and history of aggressiveness, suicidal ideation and suicide attempt from the patients were recorded. Psychiatric diagnosis was based on the ICD-10 classification [17]. Behavioral data included contact with animals, foreign traveling, frequency of meat consumption, type of meat consumed (pork, beef, goat, lamb, boar, chicken, turkey, pigeon, duck, rabbit, venison, squirrel, horse, opossum, or other), consumption of raw or undercooked meat and dried or processed meat (ham, sausages or chorizo), drinking untreated water or unpasteurized milk, consumption of unwashed raw vegetables and fruits, frequency of eating away from home (in restaurants or fast food outlets), contact with soil (gardening or agriculture), and type of flooring at home.

### Serological detection of *T. gondii* antibodies

Sera from participants were analyzed for anti-*T. gondii* IgG antibodies with the commercially available enzyme immunoassay kit “*Toxoplasma* IgG” (Diagnostic Automation Inc., Calabasas, CA, USA). This test allows determining the presence and levels of IgG antibodies. A positive result was considered when a value of equal to or higher than 8 IU/ml of specific anti-*T. gondii* IgG antibody was obtained. All sera were further analyzed for anti-*T. gondii* IgM antibodies by the commercially available enzyme immunoassay “*Toxoplasma* IgM” kit (Diagnostic Automation Inc., Calabasas, CA, USA). All tests were performed following the instructions of the manufacturer.

### Statistical analysis

Data was analyzed with the following software: Microsoft Excel 2010, Epi Info version 7 (Centers for Disease Control and Prevention: http://wwwn.cdc.gov/epiinfo/) and SPSS version 15.0 (SPSS Inc. Chicago, Illinois). For calculation of the sample size, we used a 95% confidence level, a power of 80%, a 1:1 proportion of cases and controls, a reference seroprevalence of 6.1% [15] as the expected frequency of exposure in controls, and an odds ratio of 3.0. The result of the sample size calculation was 149 cases and 149 controls. The paired student’s *t* test was used to compare age values among the groups. The McNemar’s test was used for comparison of the frequencies of seropositivity to *T. gondii* between cases and controls. The initial evaluation of the association between the seropositivity to *T. gondii* and the characteristics of the patients was performed with the Pearson’s chi-square test and the Fisher exact test (when values were small). Variables with a *P* value ≤0.10 obtained in the bivariate analysis were further analyzed to determine their association with *T. gondii* infection by multivariate analysis. Odds ratios (OR) and 95% confidence intervals (CI) were calculated by means of the backward stepwise regression method. We used the Hosmer-Lemeshow goodness of fit test to assess the fitness of the regression model. Statistical significance was set at a *P* value of less than 0.05.

### Ethics aspects

This study was approved by the Ethical Committee of the Institute for Scientific Research “Dr. Roberto Rivera Damm”, Juárez University of Durango State. The purpose and procedures of the study were explained to all participants. In addition, a written informed consent was obtained from each participant.

## Results

Anti-*T. gondii* IgG antibodies were present in 15 (10.1%) of 149 cases and in 14 (9.4%) of 149 controls. There was not difference in the seroprevalence of *T. gondii* infection among case control pairs (OR = 3.0; 95% CI: 0.12-73.64; *P* = 1.0). Of the 15 anti-*T. gondii* IgG positive cases, 13 (8.7%) had anti-*T. gondii* IgG antibody levels higher than 150 IU/ml, and 2 (1.3%) between 8 to 99 IU/ml. In contrast, of the 14 anti-*T. gondii* IgG positive controls, 7 (4.7%) had anti-*T. gondii* IgG antibody levels higher than 150 IU/ml, 1 (0.7%) between 100 to 150 IU/ml, and 6 (4.0%) between 8 to 99 IU/ml. The frequency of high (>150 IU/ml) levels of anti-*T. gondii* IgG antibodies was similar among cases and controls (*P* = 0.24). Anti-*T. gondii* IgM antibodies were found in 11 (7.4%) of the 149 cases and in 16 (10.7%) of the 149 controls (*P* = 0.31). Anti-*T. gondii* IgG antibodies were detected in 1 (3.8%) of 26 female cases and in 3 (11.5%) of 26 female controls (*P* = 0.60). While anti-*T. gondii* IgG antibodies were detected in 14 (11.4%) of 123 male cases and in 11 (8.9%) of 123 male controls (*P* = 0.52). The frequency of high (>150 IU/ml) anti-*T. gondii* IgG antibody levels was similar in male (12/123: 9.8%) and female (1/26: 3.8%) cases (*P* = 0.46).

With respect to the socio-demographic characteristics in cases, none of the variables studied (age, gender, birthplace, residence, occupation, educational level and socioeconomic status) had a *P* value ≤0.10 by bivariate analysis (Table 1).

**Table 1:** **Socio-demographic characteristics of psychiatric patients with psychoactive substance use and seroprevalence of** ***T. gondii*** **infection**

		**Prevalence of**		
	**Subjects tested**	***T. gondii*** **infection**		***P***
**Characteristic**	**No.**	**No.**	**%**	**value**
Age groups (years)				
30 or less	51	3	5.9	0.33
31-50	73	10	13.7	
>50	25	2	8.0	
Sex				
Male	123	14	11.4	0.47
Female	26	1	3.8	
Birth place				
Durango State	126	11	8.7	0.25
Other Mexican state or abroad	23	4	17.4	
Residence				
Durango State	142	15	10.6	1
Other Mexican state	7	0	0.0	
Residence area				
Urban	121	13	10.7	0.58
Suburban	22	1	4.5	
Rural	6	1	16.7	
Educational level				
No education	2	0	0.0	0.2
1-6 years	37	7	18.9	
6-12 years	90	6	6.7	
>12 years	20	2	10.0	
Occupation				
Laborer	96	11	11.5	0.57
No laborer	53	4	7.5	
Socio-economic level				
Low	55	4	7.3	0.63
Medium	93	11	11.8	
High	1	0	0.0	

Of the clinical characteristics in cases, seropositivity to *T. gondii* was not associated with the area of attention (outpatients or inpatients), psychiatric diagnosis, evolution time, response to treatment, presence of other diseases, history of lymphadenopathy, blood transfusions, transplantation and surgeries, presence of frequent headaches, dizziness, and impairments in vision, hearing, memory and reflexes, or obstetric history in women. In contrast, seropositivity to *T. gondii* was negatively associated with aggressiveness, suicidal ideation and suicide attempts by bivariate analysis (Table 2). The prevalence of high (>150 IU/ml) anti-*T. gondii* IgG antibody levels was also lower in patients with aggressiveness (5/99, 5.1%) than in those (8/50, 16%) without aggressiveness (*P* = 0.03), and in patients with suicide ideation (5/99, 5.1%) than in those (8/50, 16%) without suicide ideation (*P* = 0.03). The frequency of high (>150 IU/ml) anti-*T. gondii* IgG antibody levels was lower (but not statistically significant) in patients with suicide attempt (2/57, 3.5%) than in those (11/92, 12%) without suicide attempt (*P* = 0.13).

**Table 2:** **Clinical characteristics of psychiatric patients with psychoactive substance use and seroprevalence of** ***T. gondii*** **infection**

		**Prevalence of**		
	**Subjects tested**	***T. gondii*** **infection**		***P***
**Characteristic**	**No.**	**No.**	**%**	**value**
Patient group				
Inpatients	98	12	12.2	0.22
Outpatients	51	3	5.9	
Psychiatric diagnosis				
F10 (use of alcohol)	38	3	7.9	0.89
F12 (use of cannabinoids)	8	1	12.5	
F13 (use of sedative hypnotics)	7	1	14.3	
F14 (use of cocaine)	1	0	0.0	
F15 (use of other stimulants, caffeine)	3	1	33.3	
F17 (use of tobacco)	10	1	10.0	
F19 (use of multiple drugs)	82	8	9.8	
Subtype of disorder				
F1x.0 (acute intoxication)	2	0	0.0	0.87
F1x.1 (harmful use)	28	2	7.1	
F1x.2 (dependence syndrome)	118	13	11.0	
F1x.5 (psychotic disorder)	1	0	0.0	
Evolution time				
<10 years	79	7	8.9	0.8
10-20 years	48	5	10.4	
>20 years	22	3	13.6	
Response to treatment				
Good	85	8	9.4	0.93
Regular	53	6	11.3	
Bad	11	1	9.1	
Other psychiatric disease				
Yes	77	10	13.0	0.22
No	72	5	6.9	
Aggressiveness				
Yes	99	6	6.1	0.02
No	50	9	18.0	
Suicide ideation				
Yes	99	5	5.1	0.004
No	50	10	20.0	
Suicide attempt				
Yes	57	2	3.5	0.03
No	92	13	14.1	
Number of suicide attempts				
1-3	45	2	4.4	1
>3	12	0	0.0	
Time of last suicide attempt				
<6 months ago	26	1	3.8	1
≥6 months ago	31	1	3.2	

A selection of behavioral variables in cases and controls and their association with *T. gondii* seropositivity is shown in Table 3. Of the behavioral characteristics in cases, the variables cats in the neighborhood, dogs at home, consumption of meat from boar, duck, opossum, and armadillo, eating unwashed raw vegetables, and soil flooring at home had *P* values ≤ 0.10 by bivariate analysis. Other behavioral characteristics including cats at home, cleaning cat excrement, traveling, consumption of pork, beef, venison, lamb, or meat from goat, chicken, turkey, pigeon, or squirrel, frequency of meat consumption, consumption of dried or processed meat, drinking unpasteurized milk or untreated water, consumption of unwashed raw fruits, and contact with soil had *P* values > 0.10. Multivariate analysis of clinical and behavioral characteristics of cases with *P* values ≤ 0.10 in the bivariate analysis showed that seropositivity to *T. gondii* was positively associated with consumption of opossum meat (OR = 10.78; 95% CI: 2.16-53.81; *P* = 0.003) and soil flooring at home (OR = 11.15; 95% CI: 1.58-78.92; *P* = 0.01), and negatively associated with suicidal ideation (OR = 0.17; 95% CI: 0.05-0.64; *P* = 0.008). The result of the Hosmer-Lemeshow test (*P* = 0.96) indicated a good fit of our regression model.

**Table 3:** **Bivariate analysis of selected putative risk factors for infection with** ***T. gondii*** **in psychiatric patients with psychoactive substance use and controls**

	**Patients**	**Prevalence of**			**Controls**	**Prevalence of**		
	**tested**	***T. gondii*** **infection**		***P***	**tested**	***T. gondii*** **infection**		***P***
**Characteristic**	**No.**	**No.**	**%**	**value**	**No.**	**No.**	**%**	**value**
Cats at home								
Yes	83	11	13.3	0.14	74	9	12.2	0.25
No	66	4	6.1		75	5	6.7	
Cats in the neighborhood								
Yes	99	13	13.1	0.08	119	12	10.1	0.73
No	50	2	4		30	2	6.7	
Dogs at home								
Yes	124	15	12.1	0.07	121	11	9.1	0.72
No	25	0	0		28	3	10.7	
Birds at home								
Yes	72	10	13.9	0.13	50	5	10	1
No	77	5	6.5		99	9	9.1	
Traveled abroad								
Yes	55	3	5.5	0.15	52	5	9.6	1
No	94	12	12.8		97	9	9.3	
Boar meat consumption								
Yes	34	6	17.6	0.1	22	0	0	0.22
No	115	9	7.8		127	14	11	
Pigeon meat consumption								
Yes	30	5	16.7	0.18	23	4	17.4	0.23
No	119	10	8.4		125	10	8	
Duck meat consumption								
Yes	33	7	21.2	0.02	24	5	20.8	0.05
No	116	8	6.9		125	9	7.2	
Opossum meat consumption								
Yes	10	5	50	0.001	2	1	50	0.18
No	139	10	7.2		147	13	8.8	
Armadillo meat consumption								
Yes	10	5	50	0.001	4	1	25	0.32
No	139	10	7.2		145	13	9	
Iguana meat consumption								
Yes	14	3	21.4	0.15	5	1	20	0.39
No	135	12	8.9		144	13	9	
Degree of meat cooking								
Raw or undercooked	11	2	18.2	0.3	20	2	10	1
Well done	138	13	9.4		127	12	9.4	
Unwashed raw vegetables								
Yes	53	2	3.8	0.05	48	4	8.3	1
No	96	13	13.5		101	10	9.9	
Untreated water								
Yes	122	10	8.2	0.15	96	10	10.4	0.77
No	27	5	18.5		53	4	7.5	
Floor at home								
Ceramic or wood	76	5	6.6	0.009	61	5	8.2	0.72
Concrete	66	7	10.6		81	8	9.9	
Soil	7	3	42.9		6	0	0	

## Discussion

There are no reports on the association of *T. gondii* infection with mental and behavioral disorders due to psychoactive substance use. Therefore, through an age- and gender matched case-control study design we sought to determine such association in patients attended in a psychiatric hospital in northern Mexico. The seroprevalence of anti-*T. gondii* IgG and IgM antibodies and anti-*T. gondii* antibody levels were similar in patients than in the general population. Thus, our results suggest that mental and behavioral disorders due to psychoactive substance use are not associated with infection with *T. gondii*. Whether *T. gondii* infection might predispose to drug use or whether drug use might predispose to *T. gondii* infection is unknown. The 10.1% seroprevalence of *T. gondii* infection found in patients suffering from mental and behavioral disorders due to psychoactive substance use is comparable to the 7.4% seroprevalence of *T. gondii* infection reported in healthy blood donors in Durango City [18] but it is lower than that (21.1%) reported in waste pickers [19] and inmates [20]. Differences in contributing factors for infection among the groups may explain the differences in the seroprevalences. Altogether, results indicate that mental and behavioral disorders due to psychoactive substance use do not predispose to infection with *T. gondii*. In contrast, high seroprevalence (18.2%) of *T. gondii* infection has been reported in psychiatric patients [10], and therefore, the low seroprevalence (10.1%) found in the present study was unexpected. Reported contributing factors for *T. gondii* infection in psychiatric patients include sexual promiscuity, consumption of unwashed raw fruits, and history of surgery [10]. These factors were not associated with *T. gondii* infection in the present study. The underlying mechanisms mediating a potential association of *T. gondii* infection with mental and behavioral disorders due to psychoactive substance use have not been elucidated. Infection with *T. gondii* may lead to behavioral changes in humans and animals and these changes have been reviewed recently [21,22]. The seroepidemiology of *T. gondii* infection among drug addicts has been poorly explored. Seroprevalence of *T. gondii* infection in intravenous drug addicts was 7.7% in Vietnam [23] and 47.6% in Spain [24]. However, these studies did not provide information about a correlation of *T. gondii* infection with mental and behavioral disorders due to psychoactive substance use.

We assessed a number of putative risk factors for *T. gondii* exposure. However, none of the socio-demographic characteristics of patients was associated with *T. gondii* seropositivity. In contrast, multivariate analysis showed that *T. gondii* exposure was positively associated with consumption of opossum meat and living in a house with soil flooring. The epidemiological link of consumption of opossum meat and *T. gondii* exposure is highly feasible since 16.6% of native opossums (*Didelphis virginiana*) in Durango were found positive for anti-*T. gondii* antibodies by using the modified agglutination test [25]. In addition, seropositivity to *T. gondii* has been found in several species of opossums [26]. Recently, isolation of *T. gondii* was obtained in a black-eared opossum (*Didelphis aurita*) from Brazil [27]. Of note, seroprevalence of *T. gondii* infection was also associated with consumption of armadillo meat by bivariate analysis. However, this association did not resist the multivariate analysis. On the other hand, the association of *T. gondii* seroprevalence with living in a house with soil flooring found in the present study confirms previous observations in pregnant women in urban [28] and rural [29] Durango, Mexico. A contamination of soil floors at home with parasite oocyst might have occurred in these cases.

Of the clinical characteristics, *T. gondii* exposure was not associated with any specific type or subtype of mental and behavioral disorders due to psychoactive substance use. Intriguingly, aggressiveness, suicidal ideation and suicide attempts were negatively associated with seropositivity to *T. gondii* by bivariate analysis. Further analysis by logistic regression showed that *T. gondii* exposure was negatively associated with suicidal ideation. This result was unexpected since *T. gondii* seropositivity has been associated with suicide attempts [30,31]. In a recent study in psychiatric patients in Durango City, we found an association of high anti-*T. gondii* IgG levels with suicide attempts [32]. It is not clear why *T. gondii* exposure was negatively associated with suicidal ideation in patients suffering from mental and behavioral disorders due to psychoactive substance use in this study. Differences in the populations among the studies might account for explaining the apparently conflicting results. Depression rate was higher in the previously studied population than in patients in the present study. In addition, in the previous study, we sampled patients with a number of psychiatric disorders while in the present study we only sampled patients suffering from mental and behavioral disorders due to psychoactive substance use. It raises a question whether psychoactive substance use influences the rate of suicide behavior in *T. gondii* infected patients. Remarkably, both *T. gondii* infection and psychotropic drugs influence the dopaminergic pathways. Infection with *T. gondii* has been associated with an increase in dopamine in brain [33]. Most drugs of abuse increase the dopamine neurotransmission [34]. In contrast, a reduced dopamine synthesis in marihuana users has been found [35]. On the other hand, dopamine and serotonin have been involved in suicide behavior and aggression [36]. Therefore, alterations in neurotransmitters as dopamine due to *T. gondii* infection and substance use could be influencing the rate of aggressiveness and suicide behavior in our patients. Both substance use [37] and high levels of anti-*T. gondii* IgG antibodies [32] have been associated with suicide attempts in psychiatric patients in our region. However, the negative association of *T. gondii* infection with suicide ideation found in patients suffering from mental and behavioral disorders due to psychoactive substance use suggests a protective effect of *T. gondii* against suicide ideation in these patients. Interestingly, a negative effect of latent toxoplasmosis on the suicide-associated burden in non-European countries was recently reported [38]. Most of the studied patients in the current study used several drugs but marihuana use was common among them. It raises the question whether the reduced dopamine synthesis in marihuana users might be somewhat overcame for an increase in dopamine production by *T. gondii* resulting in normal levels of dopamine and low rates of suicide ideation. On the other hand, it is unknown whether an overproduction of dopamine by both *T. gondii* infection and some psychotropic substances might also lead to a reduction of suicide ideation rates. The role of *T. gondii* in the production of neurotransmitters and their interactions has been scantly studied. Apart from dopamine, other neurotransmitters might play a role in suicide behavior. Low cerebrospinal fluid concentrations of 5-hydroxyindolacetic acid has been associated with suicidal behavior [39,40]. Whether production of dopamine (and perhaps other neurotransmitters) by *T. gondii* is compensating excitatory or inhibitory effects of neurotransmitters associated with suicide behavior should be elucidated. Infections with *T. gondii* or HIV/*T. gondii* co-infections in intravenous drug users have correlated with altered serum cytokine levels including increased levels of TNF-α, IL-6 and IL-12 [41]. Further studies with a different study design to assess the negative association of *T. gondii* exposure with suicidal behavior in patients suffering from mental and behavioral disorders due to psychoactive substance use is needed.

In the present study, analysis of the association of *T. gondii* infection with the characteristics of the patients was based on results of anti-*T. gondii* IgG antibodies. In fact, anti-*T. gondii* IgG antibodies appear very early after infection [42]. Results of anti-*T. gondii* IgM antibodies were not included in the analysis because of a number of limitations for the diagnosis of *T. gondii* infection. Firstly, tests for detection of anti-*T. gondii* IgM antibodies may have a high rate of false positive results caused by limitations in test specificity [43]. Therefore, interpretation of the increased IgM seroprevalence found in the current study should be taken with care. Secondly, the absence of anti-*T. gondii* IgM in subjects with anti-*T. gondii* IgG antibodies indicates a chronic infection but the presence of the IgM marker does not necessarily indicate an acute infection. Anti-*T. gondii* IgM antibodies are detectable early after infection and can persist for prolonged periods of time after infection [2,43]. Therefore, seropositivity to IgM alone is not considered an acceptable diagnostic criterion for acute infection.

## Conclusions

We conclude that infection with *T. gondii* is not associated with mental and behavioral disorders due to psychoactive substance use. This is the first report of a positive association of *T. gondii* exposure with consumption of opossum meat. Further studies to elucidate the role of *T. gondii* infection in suicidal ideation and behavior are needed to develop optimal strategies for the prevention of infection with *T. gondii*.
